# Remarks upon Compound Dislocation of the Elbow-joint—Case, Followed by Tetanus—Amputation—Death upon the Sixth Day

**Published:** 1869-01

**Authors:** Theophilus Mack

**Affiliations:** St. Catherines, Ontario


BUFFALO

### Medical and Surgical Journal.

VOL. VIII. JANUARY, 1869. No. 6.

### Original Communications.

ART. I.—*Remarks upon Compound Dislocation of the Elbow-joint—Case, followed by Tetanus—Amputation—Death upon the sixth day.* By Theophilus Mack, M. D., *St. Catherines, Ontario.*

This extremely rare accident is but feebly or cursorily noticed by systematic surgical writers, while those who have confined themselves more exclusively to the affections of joints, with one exception, have not established any satisfactory course to guide the practitioner in the presence of such a casualty. The violent tearing open of any articulation, and displacement of the bones composing it, is at all times a formidable affair, but especially so in the ginglymoid joints of the extremities, and although several remarkable recoveries have occurred in the case of the knee-joint, very few are recorded of the elbow-joint, so few that it is fair to infer that although this dislocation is undoubtedly unusual, it is yet more rarely given to the profession through the medium of the press. The only cases I have been able to lay my hands upon are the following:

Samuel Cooper states, "in a modern publication, an instance of a dislocation of the heads of the radius and ulna backward is related, where the lower end of the humerus protruded through the integuments, and as it could not be reduced, it was sawed off.

The patient, a boy, recovered the full use of his arm." (Evans' Pract. Obs. on Fracture, Comp. Disloc., etc.)

Sir Astley Cooper gives a case in the words of the dresser, Mr. Samuel White, in which "the condyles of the humerus were thrown inwards through the skin; the articulating surface receiving the sigmoid cavity of the ulna being completely exposed to view; the ulna was dislocated backwards, and the radius outwards; the lateral and capsular ligaments were torn asunder, with extensive laceration of the parts about the joint, but the artery and nerve remained perfectly free from injury." The displacement was remedied, the wound in the soft parts properly dressed, a splint of pasteboard was applied, securing the arm in a semi-flexed position. On the third day v. s. to the extent of ten ounces was performed, and the boy, aged 13, made a good recovery in less than two months.

In the *Medical Times and Gazette*, July 5th, 1856, a case is reported of a boy aged 12 years, under the care of Mr. Curling: "the lower end of the humerus was found to be protruding to the extent of three inches through a crescentic wound of about two inches in length on the inner side of the elbow. The olecranon and head of the radius are very prominent backwards and outwards. There is great injury of the soft parts; through the wound the biceps and brachialis anticus appear lacerated, and the median and external cutaneous nerves are stretched tightly over the anterior surface of the protruded bone, but are not torn." The dislocation being reduced a well padded angular splint was applied, the wound united by suture and wet lint applied, etc. Destructive inflammation resulting in exfoliation of about half an inch square of the external condyle ensued, and the boy recovered in about three months with a very limited amount of motion in the joint.

At the Salford Royal Hospital, under the care of Mr. Windsor, a boy aged 14, on 18th March, 1856, was admitted. "In front of the left elbow-joint there is a nearly transverse wound through which the whole of the inferior extremity of the humerus has protruded; for about two inches of its extent, the median nerve is exposed, and thrown forwards by the humerus, on which it rests; the brachial artery is exposed to the extent of half an inch, and is felt pulsating on the inner side of the wound; there is no hæmorrhage at present. The forearm is somewhat swelled and echymosed; both the radius and ulna are fractured near their middle, (simple fracture.") The projecting portion of the humerus was sawn off, the sharp edges rounded and reduction effected. Recovery took place in about two months.

These cases leave an ambiguity greater even than usual; as to the practice to be deduced from them, two recovered without loss of bony tissue, and two recovered with. I am led to surmise that there is so little for self-gratulation in the management of such cases, that few men have experienced any desire to publish results so far from brilliant. I am also inclined to believe that so much injury to nerve tissue occurs, that severe constitutional shock or tetanus is more likely to follow compound dislocation of the elbow than in the majority of compound dislocations. In most other joints the most formidable consequences consist in the pathological changes of the synovial membrane, ulceration of cartilage, pyemia, etc.

In compound dislocation of the elbow the protruding bone is usually the humerus; there is first laceration of the integuments of the front and inner side of the joint. Second; the fibres of the brachialis anticus and the tendon of the biceps. Third; the two lateral ligaments and the capsule of the joint. There may be also injury to a greater or less degree to the following important parts: The broad ligament from the forepart of the humerus to the coronoid process and orbicular ligament, the musculo cutaneous and median nerves, the brachial artery and veins. Externally there will be placed upon the stretch, the supinator longus, the extensor carpi radialis longior, the musculo-spinal nerve and recurrent radial artery; the ulnar nerve in relation with the internal lateral ligament, and the posterior ligament, connecting the back of the humerus between the condyles with the base of the olecranon. The synovial membrane is also extensively reflected upon the other ligaments and surrounds the head of the radius, forming an articulating sac between it and the lesser sigmoid notch.

Druitt recommends generally that compound dislocations should be reduced; the end of the bone to be sawed off, if it render reduction difficult. The evils to be dreaded are the consequences of inflammatory action generally; nothing is said of particular compound dislocations.

Ferguson recommends in the less severe cases "to try the chance of saving the limb, without or with excision," etc.

Gross recommends amputation, "*if the joint be extensively opened, the muscles torn and the bones seriously involved;*" to avoid the danger from protracted suppuration and ulceration; under more favorable auspices, excision of the ends of the injured bones.

Samuel Cooper reprobates the practice of resection, recommends amputation when unavoidable, but urges reduction and conservatism, and speaks only of the dangers from suppurative inflammation.

Erichsen alludes only to the perils from destructive inflammatory action, and advises in compound dislocations of the upper extremity when the injury is not very extensive, replacement of the bone followed by cold irrigation and antiphlogistic treatment.

R. M., aged 13 years, fell from a tree, a distance of a few feet, and in the act of falling a heavy branch struck him upon the right arm. No more circumstantial or distinct account of the accident can be elicited. After the fall he walked a distance of about four hundred yards. Upon examination the lower end of humerus was discovered thrust through an oblique laceration upon the inner surface of the elbow-joint to the extent of more than two inches; the olecranon could be felt positively, the head of the radius outwards. It was evident that all the ligaments having attachments to the condyles were ruptured as well as the tendon of the biceps and the fibres of the brachialis anticus; it was also probable that the median nerve had been injured. Amputation appeared to be the only resource, and expressed my apprehensions to the boy's sisters that lock-jaw was to be expected without the adoption of that measure, but as both parents were far from home and a certainty was expressed by the other members of the family that the operation would not be permitted, or any operative proceeding, I was obliged to entertain the alternative of replacing the bone; this was effected through the narrow opening in the integuments by the leverage of the handle of a silver spoon, with extension of the forearm sexi-flexed, through the assistance of my brother, Dr. F. L. Mack. The wound having been united by silver sutures, the arm was carefully and loosely secured to an angular splint, and carbolic acid dressing applied with lint covered with tin-foil.

At 9 P. M., 10th October, a few hours after the injury, the boy appeared easy, and he was ordered one-eighth of a grain of morphine, combined with twice as much tartar emetic, every four hours.

11th, 9 A. M. The little patient slept about three hours during the night, occasional spasmodic action in the wounded limb, not much pain, the intervals between the doses of medicine to be reduced to two hours. Bowels acted at noon, spasms abated in frequency towards night, but were attended with more pain, feverish, joint much swollen, complains of pain in back of neck.

12th, 9 A. M. Slept well most of the night, spasms relieved, removed splint, arm much swollen; it was laid upon a cushion; slight suppuration at the lower end of the laceration. Mother arrived to-day. Dressing as before; omit morph. and ant., beef-tea and wine; Seidlitz to move the bowels, at 6 P. M.

13th. Rested well, no spasms, bowels not opened, a cathartic of hyd. chlor. rhei and bicarb. soda was administered.

14th. Passed a restless night, pulse 120, tense febrile symptoms increasing, bowels not opened. At 2 P. M. bowels acted freely, after enema of ol., ricine and terebinth, beef-tea and wine, etc., given freely. 7 P. M. very restless, suppuration increasing, spasmodic contractions of the extremity have returned, slight stiffness complained of in the lower jaw. Indian hemp was freely exhibited all night, and frictions of chloroform liniment used along the spine. Dr. E. Goodman in consultation.

15th. All the symptoms much aggravated, stiffness of back of neck and inability to open the mouth; the friends having been urged to allow amputation of the arm, the father was telegraphed to for permission. At 1 P. M., assisted by Drs. Goodman, Comfort, Ville, Sullivan and F. L. Mack, amputation at the lower third of arm by a double flap was performed under perfect anæsthesia, the wound was secured by silver sutures, and a solution of sulphate of morphia applied on lint. Chapman's spinal ice-bag was kept applied. Answer to telegraph was received urging a delay of operation, unless gangrene had commenced.

For a few hours after the operation a slight amelioration in the symptoms took place, but severe opisthotonos soon supervened, the cannabis was given every half hour until unmistakable physiological effects were produced, and chloroform was freely inhaled according as the severity of the spasms demanded. At 1 P. M. on the 16th, the face appeared anxious, pulse tense and quick, respiration frequent and difficult, tetanic spasms constant when effect of chloroform diminishes; from this time to the hour of death, at 6 A. M., the agony of the little sufferer was extreme.

In this case, from the very outset, I had an instinctive dread of tetanus, and amputation upon the spot appeared to me evidently to be the only expedient to avert the formidable catastrophe. Had I even, with the consent of the friends, been able to operate with the conflicting authorities, and from the *charité de metier*, I should, in any event, have been exposed to the charge of *nimia diligentia*, and it is to give weight to the decided opinion of my old mentor and friend, F. H, Hamilton, that I place this unfortunate case upon record. Professor Hamilton is the only author who has condemned the reduction of compound dislocation upon proper grounds, viz.: "the violent strain of the muscles, tendons, and other soft tissues."

In all cases of compound dislocation of the elbow-joint the two following rules should be considered absolute:

1st.—If injury to the nerves or artery has been done, amputate as soon as the pulse will warrant the operation.

2d.—In cases where amputation appears unnecessary, let reduction be effected after resection, never without.

